# Abstract of Proceedings of the Buffalo Medical Association

**Published:** 1866-09

**Authors:** T. M. Johnson

**Affiliations:** Sec’y.


ART. IV—*Abstract of Proceedings of the Buffalo Medical Association.*

TUESDAY EVENING, July 3d, 1866.

The Society was called to order by the President at the usual hour. Present—Drs. Gould, Wetmore, Samo, Congar, Boardman, Taft and Camerling.

There being no voluntary communications offered, the President suggested the propriety of taking into consideration the subject of revising, if necessary, and printing the Constitution and By-Laws in a form convenient for the use of the members.

Considerable discussion was had upon the subject, in which most of the members present participated. No definite conclusion having been arrived at as to the best course to pursue in the matter, the President proposed that revising and printing the Constitution and By-Laws be made the special subject for the next meeting.

Report on prevailing diseases being called for the members present were unanimous in the conclusion that the health of the city is unusually good, and that there is no special disease prevailing.

Adjourned. T. M. JOHNSON, Sec'y.

---

TUESDAY EVENING, August 7, 1866.

The President and Secretary were present—remained until 8 o'clock, when a quorum not being present, the rooms were closed by the President.

---

Authentic advices received at Alexandria from Djeddah report that cholera has broken out among the returning pilgrims from Mecca, and that there is great mortality among the Egyptian soldiers.

